# A Case Report of Chondrosarcoma of Temporomandibular Joint

**DOI:** 10.1007/s12070-021-02483-2

**Published:** 2021-03-08

**Authors:** Hage Ampu, Tanya Singh, Sunil Kumar, H. P. Singh, Shalini Bhalla

**Affiliations:** 1 Department of ORL-HNS, King George Medical University, Lucknow, India; 2 Department of ORL, Career Institute, Lucknow, India; 3 Department of Pathology, King George Medical University, Lucknow, India

**Keywords:** Chondrosarcoma, Temporomandibular joint, TMJ

## Abstract

In this case report we describe a rare case of chondrosarcoma of the Temporomandibular joint in a 70 years old female who presented with a right preauricular swelling, trismus and neuralgic pain. On examination, firm and tender swelling was noted in the right preauricular region. CT Scan revealed 3.48 × 3.0 cm size mass lesion in the region of mandibular condyle and extending into the right temporomandibular joint space. The cytopathological report was suggestive of chondroid malignancy. The tumor was excised and histopathological examination showed large sheets of atypical tumor cells with cartilaginous matrix and diagnosis of a well differentiated Chondrosarcoma was confirmed. Post-surgical resection, patient remains disease free at 15 months follow up.

## Introduction

Chondrosarcoma is a malignant cartilage forming tumor [1]. Chondrosarcoma is the most common bone tumor following osteogenic sarcoma, and comprises 10–20% of all primary bone tumors. It commonly occurs in pelvis, ribs, femur and humerus and is rarely seen in the head and neck region. It represents approximately 0.1% of all of head and neck neoplasms. Only 5% to 10% of chondrosarcomas occur in the head and neck, with the larynx and the maxillo-nasal region being the most common sites [2]. The occurrence of Chondrosarcoma in Temporomandibular joint is very rare [2, 3].

## Case Report

**Fig. 1:**
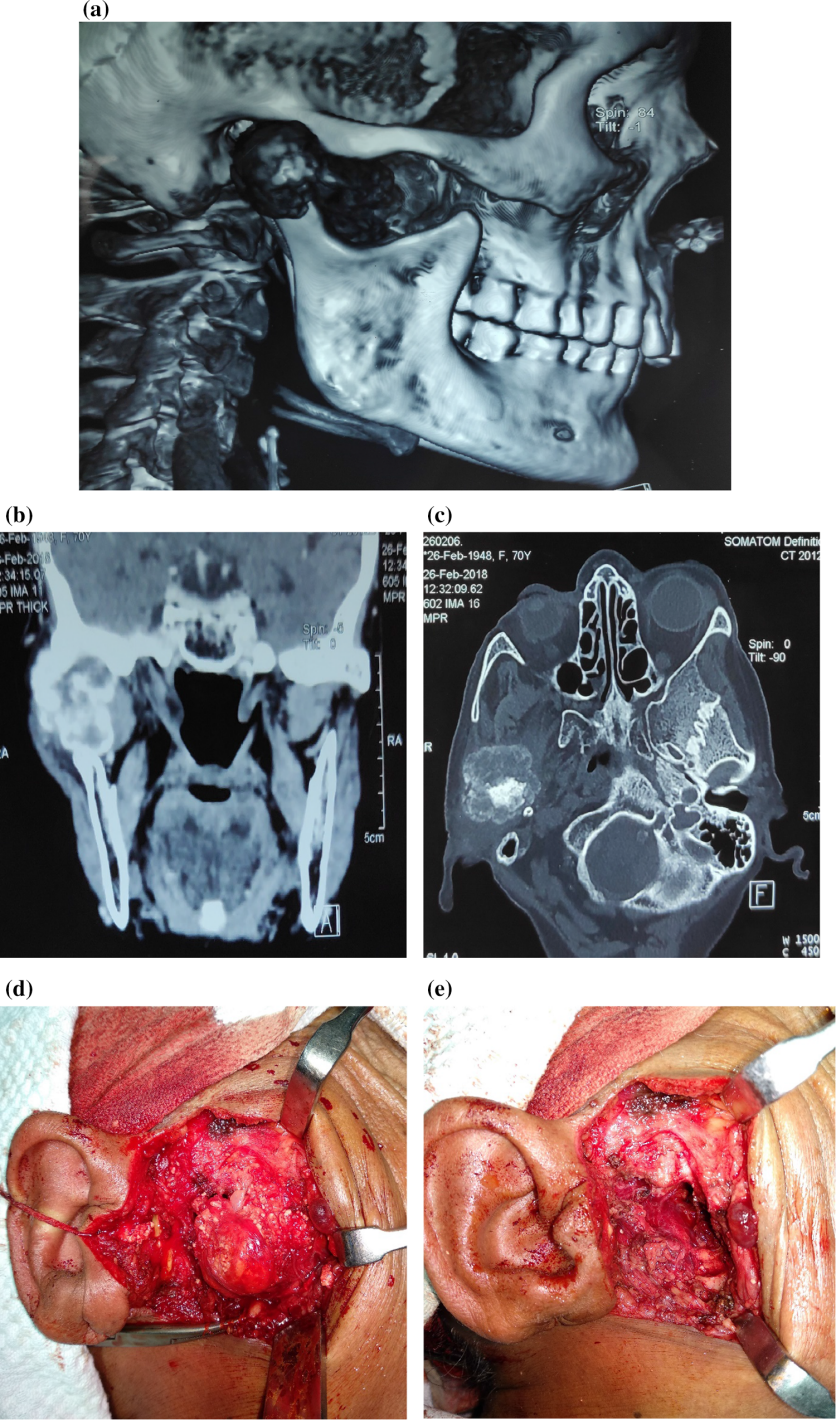

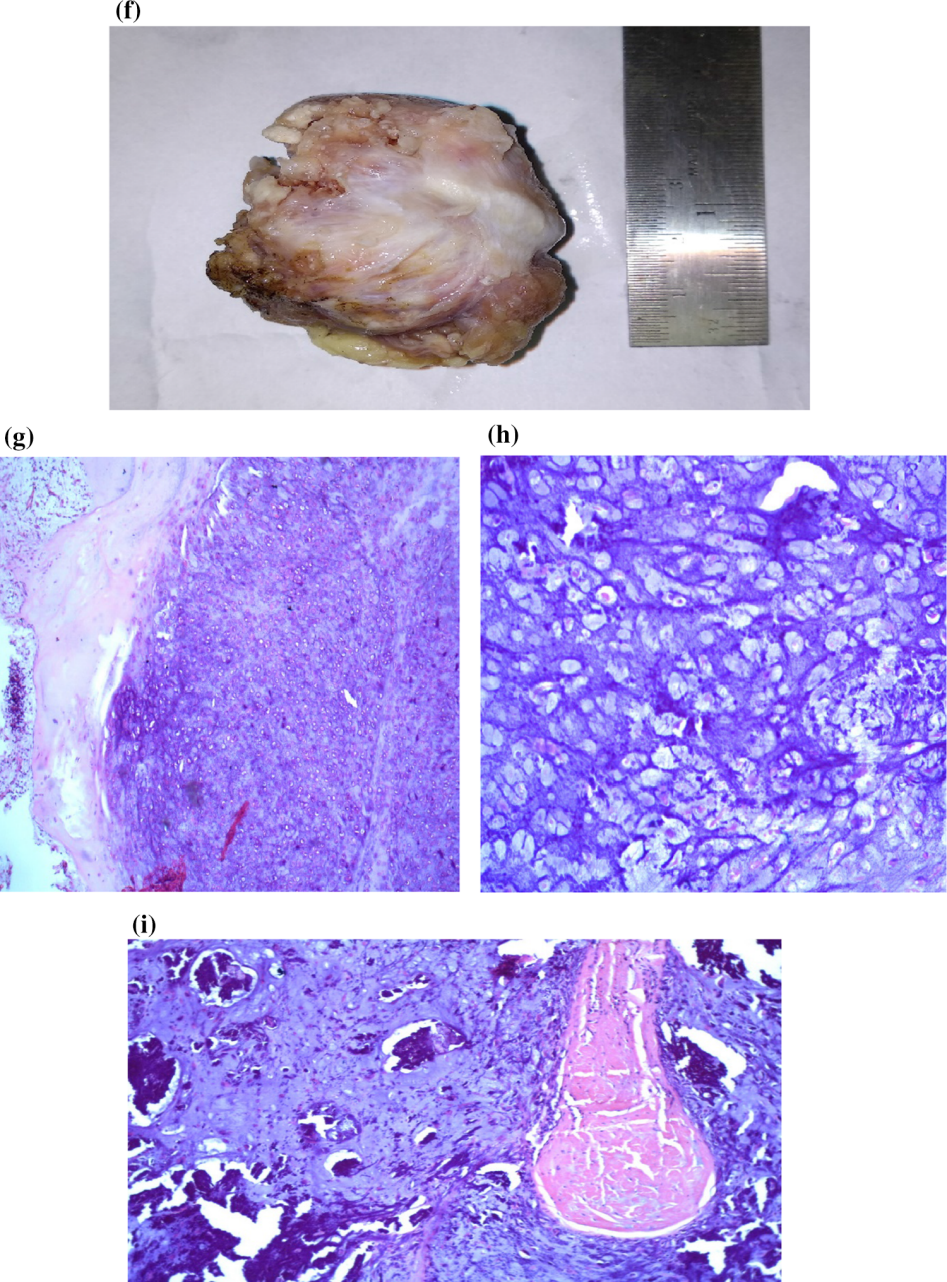
**a** Radiograph of the lateral view of skull showing a well-defined tumor involving the right temporomandibular joint. CECT head, coronal (**b**) & sagittal section. **c** Mass involving right mandibular condyle and coronoid process with multiple radiolucent areas and calcifications causing condylar deformity. **d** Well encapsulated globular tumor visualised after a preauricular dissection. **e** Defect post tumor excision. **f** Smooth, capsulated, greyish white, globular mass with underlying bone & haemorrhagic areas. **g** (H&E Stain, ×10) Cellular chondroid tumor. **h** Sheets of atypical tumor cells lying in chondroid matrix. **i** Broken fragments of mature bony trabeculae with surrounding infiltrating tumor

A 70-year-old female patient presented to our outpatient department with the complaints of a swelling in the right preauricular region and limitation of jaw movements due to pain for 4 years. The patient also complained of right sided headache and facial pain. On examination the swelling was approx. 3 × 3 cm in size, firm, tender and fixed to the underlying structure while the overlying skin appeared normal and mobile. Her mouth opening was significantly reduced up to one finger. There was no facial palsy or any cervical lymphadenopathy. Her routine blood investigations were normal. Fine needle aspiration cytology revealed calcification, chondromyxoid stroma and malignant cells with moderate pleomorphism and well-defined cytoplasm which was suggestive of Cartilaginous malignancy (possibility of chondrosarcoma). Plain radiograph of the skull (Fig. 1a) revealed a well-defined mass involving the condylar process of the right Temporomandibular joint.

CECT Head, Neck & Thorax (Fig. 1b) revealed an osteo expansile lesion with cartilaginous matrix involving condylar and coronoid process of right mandible (measuring approx. 3.1 cm × 3.5 cm × 2.3 cm), abutting the mandibular process of temporal bone superiorly. There was no evidence of osseous destruction. There was no evidence of pulmonary metastasis.

A preauricular incision was given and the tumor was resected along with a part of the ascending ramus. The mandibular fossa was found to be uninvolved.

The excised specimen (Fig. 1f) measured 4 × 4 × 3 cm with smooth and capsulated outer surface. The cut section showed greyish white areas along with areas of haemorrhage. On microscopic examination of formalin fixed, paraffin embedded sections stained with haematoxylin and eosin showed atypical tumor cells arranged in sheets with cartilaginous matrix. Individual atypical cells showed high nucleo-cytoplasmic ratio, round hyper lobated, hyperchromatic nuclei and moderate amount of cytoplasm. Binucleate and trinucleate forms were also seen. The diagnosis of well differentiated Chondrosarcoma was made.

## Discussion

Chondrosarcoma of the temporomandibular joint is a very rare occurrence [2, 3] and around 30 cases have reported till date [4]. It has been reported in patients as young as 7 years old and as old as 75 years with the mean age of presentation being 46.5 years [4] and with a slight female preponderance [3–5]. It usually presents with a preauricular swelling and painful jaw movements [5]. The diagnosis is based on clinical, radiological and Histopathological examination. Radiographically, no pathognomonic findings are associated with chondrosarcoma, although single or multiple radiolucent areas can be seen with calcifications, condylar deformity and bony erosions [3, 4, 6]. Histopathologically, the tumor has been classified into grades I, II, and III based on the frequency of mitoses, cellularity, and nuclear size [2, 5]. In our case, it was a well differentiated chondrosarcoma i.e., Grade-I tumor with moderate cellularity, hyperchromatic nuclei & occasional binucleate and trinucleate cells. Low grade chondrosarcoma has better prognosis and they rarely metastasise [2]. The most effective treatment is surgical resection [5]. Tumour grade and complete resection are the most important prognostic factors for head and neck chondrosarcoma [6, 7]. Radiotherapy is required in cases of unresectable tumors, incomplete resected tumors and in high grade tumors as post op adjuvant therapy ^(7)^. In our patient there was no clinical or radiological evidence of metastasis or local infiltration so a simple excision without neck dissection sufficed. Post-surgical excision there has been no recurrence in 15 months follow up.
